# Time-dependent phenotypical changes of microglia drive alterations in hippocampal synaptic transmission in acute slices

**DOI:** 10.3389/fncel.2024.1456974

**Published:** 2024-11-15

**Authors:** Laura Ferrucci, Bernadette Basilico, Ingrid Reverte, Francesca Pagani, Giorgia Scaringi, Federica Cordella, Barbara Cortese, Gaia De Propris, Andrea Galeone, Letizia Mazzarella, Alessandro Mormino, Stefano Garofalo, Azka Khan, Valeria De Turris, Valentina Ferretti, Paola Bezzi, Cornelius Gross, Daniele Caprioli, Cristina Limatola, Silvia Di Angelantonio, Davide Ragozzino

**Affiliations:** ^1^Department of Physiology and Pharmacology, Sapienza University, Laboratory Affiliated to Institute Pasteur Italia–Fondazione Cenci Bolognetti, Rome, Italy; ^2^Center for Life Nano and Neuro Science, Istituto Italiano di Tecnologia (IIT), Rome, Italy; ^3^IRCCS Santa Lucia Foundation, Rome, Italy; ^4^National Research Council-Nanotechnology Institute (CNR Nanotec), Rome, Italy; ^5^Department of Biology and Biotechnology, Sapienza University, Rome, Italy; ^6^Department of Fundamental Neurosciences, University of Lausanne, Lausanne, Switzerland; ^7^Epigenetics and Neurobiology Unit, European Molecular Biology Laboratory (EMBL), Monterotondo, Italy; ^8^Istituto di Ricovero e Cura a Carattere Scientifico (IRCCS) Neuromed, Pozzilli, Italy

**Keywords:** microglia, acute slices, synaptic transmission, microglia reactivity, electrophysiology

## Abstract

It is widely acknowledged that microglia actively regulate synaptic function in the brain. Remarkably, much of our understanding regarding the role of microglia in synaptic regulation is derived from studies in acute brain slices. However, it is still uncertain to what extent the preparation and maintenance of acute slices can influence microglial function and whether microglial changes may affect synaptic transmission. In this study, we examined the impact of acute slice resting time on hippocampal CA1 microglia, by assessing morphological and functional parameters at two distinct time intervals. We report that after 4 h from slicing microglia undergo morphological, functional, and transcriptional changes, including a decrease in the number of branches and in their movement speed. Furthermore, microglia acquire a reactive phenotype, characterized by increased amplitude of outward rectifying K^+^ currents, increased expression of the pro-inflammatory cytokine Tnfα and altered expression of the microglial receptors Cx3cr1 and P2y12r. We also examined time-dependent changes of excitatory synaptic transmission in CA1 pyramidal neurons from acute hippocampal slices, reporting time-dependent decrease in both amplitude and frequency of postsynaptic currents (sEPSCs), along with a decrease in spine density. Noticeably, sEPSCs amplitude decrease was absent in slices prepared from PLX5622 microglia-depleted mice, suggesting that this time-dependent effect on synaptic transmission is microglia-dependent. Our findings highlight possible causal relation between microglia phenotypic changes in the hours following slice preparation and concomitant synaptic changes, pointing to the mechanisms of acute synaptic modulation, whose understanding is crucial for unraveling microglia-neurons interplay in nature. Furthermore, they emphasize the potential issues associated with experimental time windows in ex vivo samples.

## 1 Introduction

Microglia are the immunocompetent cells of the Central Nervous System (CNS) and were originally thought to be active exclusively in case of brain damage. Studies published over the past two decades have revealed that microglia have more complex roles during brain development (Paolicelli et al., 2011; Schafer et al., 2012; Basilico et al., 2019a; Andoh and Koyama, 2021b) and in the mature brain, both in health and in pathological conditions (Sierra et al., 2014; Badimon et al., 2020; Basilico et al., 2022b). It is now recognized that microglia are constitutively necessary for proper synaptic functioning (Basilico et al., 2022b,2022a) and for the establishment of synaptic plasticity (Crapser et al., 2021; Cornell et al., 2022; Hashimoto et al., 2023); for this very reason, some authors have suggested the use of the term ‘quad-partite synapse’ (Schafer et al., 2013).

The development of the preparation of living acute brain slices (Edwards et al., 1989) has been a fundamental achievement that has enabled a major advance in the study of synaptic function and in fact numerous findings on the properties of brain circuits have been obtained through the utilization of this technique (Lein et al., 2011; Ting et al., 2014). In recent years, an enormous effort has been made to optimize the preparation of acute slices as much as possible, in order to make ex vivo recordings a viable alternative to in vivo recordings or neuronal cultures (Hájos and Mody, 2009; Buskila et al., 2014; Ting et al., 2014; Eguchi et al., 2020). Although this model remains extremely important for studying synaptic properties, the procedure and the maintenance of slices in artificial CSF may represent a potential shortcoming, due to time-dependent changes in the slice health, as well as in specific functional characteristics.

This issue might be particularly relevant when studying microglia and microglia dependent effects, as these cells are extremely dynamic and reactive and even the slightest changes in the surrounding tissue microenvironment can alter their function (Arcuri et al., 2017; Wolf et al., 2017; Woodburn et al., 2021). Noticeably, the role of microglia in the regulation of synaptic function has been studied mainly with electrophysiological techniques, making acute slices an important tool in the context of bidirectional communication between microglia and neurons.

Nonetheless, it has still to be elucidated how microglia react to slicing and in vitro maintenance of acute slices and whether microglia changes could interfere with microglia-neuronal communication, affecting synaptic transmission properties. To this aim, we used different techniques to characterize the time-dependent changes in microglia phenotype in acute slices. Moreover, through voltage-clamp of pyramidal neurons in the CA1 area of the hippocampus at different intervals from slicing, we studied the time-dependent changes on synaptic transmission in presence or absence of microglia, in order to highlight the causal role of microglia in these changes.

We report that microglia undergo time dependent phenotypical changes in acute hippocampal slices. In addition, we report that the presence of microglia is required for time dependent changes in spontaneous excitatory synaptic activity, pointing to a causal association linking microglia changes to synapse functioning.

## 2 Materials and methods

### 2.1 Animals

*Cx3cr1^+/GFP^* mice were used for the analysis of microglia morphology, for time-lapse imaging of microglial processes and for the recordings of microglia K^+^ currents. Wild type (WT) C57BL6/J mice were used for microglia isolation from acute slices and for patch-clamp recordings from hippocampal pyramidal neurons. Thy1::GFP; Cx3cr1::cre ERT2; R26CAG-tdtomato mice were used for dendritic spines analysis and microglia-neuron contacts analysis. Cre-mediated recombination was induced by a single injection of tamoxifen diluted in corn oil at 10 mg/mL (Sigma, 1 mg injected per 20 g of mouse weight) one week before the experiment. All experiments were performed on male mice of 40–50 postnatal days (P40–50).

Mice were maintained under 12-h light/dark cycle (light on at 7 a.m.) with food and water *ad libitum*. For experiments in slices from microglia-depleted mice, microglia depletion was achieved through PLX5622, a pharmacological selective inhibitor of CSF1R, essential for microglial proliferation, differentiation, and survival (Elmore et al., 2014; Basilico et al., 2022b). PLX5622 was formulated in the chow at 1200 PPM by Research Diets. Drug-free standard chow (Research Diets) was used in control experiments. Mice were fed with either control diet or PLX5622 supplemented chow in the 7 days before the experiment. PLX5622 was kindly provided by Plexxikon Inc. (Berkeley, USA).

All the procedures followed the guidelines of the national law (DL 26/2014) on the use of animals for research based on the European Communities Council Directive (2010/63/UE) and approved by the ethics committee of the Italian Ministry of Health.

### 2.2 Acute slices preparation

Acute hippocampal slices were obtained from adult mice (P40–50). Animals were decapitated under halothane anesthesia (SigmaAldrich, Milan, Italy). Brains were rapidly extracted and immersed in ice-cold ACSF, containing (in mM): KCl 2.5, CaCl_2_ 2.4, MgCl_2_ 1.2, NaH_2_PO_4_ 1.2, glucose 11, NaHCO_3_ 26, glycerol 250, continuously oxygenated with 95% O_2_ and 5% CO_2_ to maintain the physiological pH. Horizontal 250 μm thick slices were cut at 4°C, using a vibratome (DSK, Dosaka EM, Kyoto, Japan) and placed in a chamber filled with oxygenated ACSF containing (in mM): NaCl 125, KCl 2.5, CaCl_2_ 2, MgCl_2_ 1, NaH_2_PO_4_ 1.2, NaHCO_3_ 26 and glucose 10. Slices were allowed to recover at room temperature (24 ± 1°C) and were used for the experiments in two different time windows: within 4 h after slicing (*early* phase) or between 4 and 8 h after slicing (*late* phase).

### 2.3 Analysis of microglia morphology

Acute slices were obtained from *Cx3cr1*^+/*GFP*^ mice and allowed to rest for the resting intervals described above. At the end of the resting interval, the slices were placed in 4% PFA overnight and the next day incubated for 24 h in PBS at 4°C. Images of microglia from the *stratum radiatum* of the hippocampus were acquired using a confocal microscope (FV10i Olympus) with a water immersion 60x objective, with a z-step size of 1 μm. Image analysis was performed using ImageJ software (NIH). Maximal intensity projections of confocal images were analyzed to obtain a morphological reconstruction of microglia (Basilico et al., 2019a). Only cells whose bodies and processes have been entirely enclosed in the image were included in the analysis. For each cell, the soma area was measured by drawing a line around the cell body and the extent of microglial branching was quantified by measuring the area circumscribed by the ends of each process (arborization domain). Moreover, we calculated the morphological index, i.e. the ratio between the soma area and the arborization domain. At last, through skeleton analysis, we calculated the total number of microglial processes.

### 2.4 Time-lapse imaging of microglial processes

Acute slices were obtained from *Cx3cr1*^+/*GFP*^ mice and allowed to rest for the resting intervals described above. Time-lapse fluorescence acquisitions were obtained at the two different time-points using an upright microscope (Axioskope) equipped with a 40 × water immersion objective (Achroplan Carl Zeiss), a digital 12 bit CCD camera system (Sensi Cam, PCO AG, Germany) and the software Till Vision v. 4.0 (Till Photonics). Excitation of GFP was achieved at 488 nm, using a 1-nm-band width polychromatic light selector (Till Polychrome V), equipped with a 150 W xenon lamp (Till Photonics, Germany). Microglial processes movement was monitored in the CA1 *stratum radiatum* by acquiring an image every 10 s for 25 min. All processes basal movements were analyzed using a custom-written Matlab code, for correction of the minimum spatial resolution of the acquired image defined by the smallest resolvable distance between two points in an image (i.e. d = 0.61 * wavelength/numerical aperture = 0.37 mm). Briefly, each cluster of detection was set to be a minimum of 1 resolution apart from each other. Processes that endured less than 2 resolutions (0.74 mm) for more than 2 min were excluded. The customized trajectories were therefore transferred into a new coordinate system, in which the origin (*x* = 0, *y* = 0) was set to the starting position of each process and analyzed to obtain migration track length (total migration distance) and instantaneous speed. Tracking data and statistical analysis was analyzed using Origin 8.0 (Basilico et al., 2019b). Please, note that in this analysis the actual movement of microglial processes that we were able to monitor in our recordings lasted on average 6–7 min.

In addition to the basal movement of microglial processes, we analyzed the rearrangement of microglial processes in response to an extracellular source of ATP. A glass pipette containing ACSF supplemented with adenosine 5’-triphosphate magnesium salt (ATP, 3 mM; Sigma Aldrich) was placed in the *stratum radiatum* in the center of the recording field. Changes in GFP fluorescence distribution were monitored by acquiring a fluorescent image every 10 s for 50 min. The basal fluorescence was monitored for 10 min, and then Mg-ATP was pressure applied to the slices (100 ms; 8 psi) with a Picospritzer III (Parker Instrumentation). To quantify the speed of microglial processes rearrangement towards the pipette tip, we measured the increase of GFP fluorescence in a circular area centered on the pipette tip (10 μm radius). At each time point the fluorescence increase in the area was calculated as ΔF = F-F0, and then divided for F0 (ΔF/F0, where F0 is the average fluorescence before ATP puff), to normalize basal GFP fluorescence.

### 2.5 Patch-clamp recordings of microglia K^+^ currents

Acute slices were obtained from *Cx3cr1*^+/*GFP*^ mice and allowed to rest for the time intervals as described above. To study the effect of acute slicing on microglial voltage-dependent K^+^ currents, we performed a meta-analysis of previously published data (Pagani et al., 2015), reclassifying them according to the resting interval before the cell was patched. As described above, the data were classified into two intervals: within 4 h after cutting or between 4 and 8 h after cutting. Acute slices were obtained as described above. Visually identified GFP-expressing microglial cells in the CA1 *stratum radiatum* were patched and placed in a whole-cell configuration. Recordings were performed on cells located deep within the tissue, since the slicing procedure could induce microglia reactivity especially near the surface of the slice. Micropipettes with a resistance of 4–5 MΩ were filled with a solution containing (in mM): KCl 135, BAPTA 5, MgCl_2_ 2, HEPES 10 and Mg-ATP 2 (pH 7.3 adjusted with KOH, osmolarity 290 mOsm; Sigma Aldrich). Voltage-clamp recordings were carried out with an Axopatch 200A amplifier (Molecular Devices). Currents were filtered at 2 kHz, digitized (10 kHz) and collected with Clampex 10 (Molecular Devices); analysis was conducted off-line with Clampfit 10 (Molecular Devices). The current/voltage (I/V) relationship of each cell was established by applying voltage steps from −170 to +70 mV (V = 10 mV) for 50 ms, maintaining the cell at −70 mV between steps. The resting membrane potential and membrane capacitance were measured at the beginning of the recording. The amplitude of the outward and inward rectifier K^+^ current was assessed after subtraction of the leakage current by a linear fit of the I/V curve between −100 and −50 mV. Cells were considered as expressing outwardly rectifying K^+^ current when the I/V relationship exhibited rectification above -30 mV and the amplitude measured at 0 mV was at least 10 pA, after subtraction of leakage; similarly, cells showing inward rectification below −100 mV were classified as expressing inwardly rectifying K^+^ current when the amplitude of the subtracted current was at least 5 pA at −150 mV.

### 2.6 Isolation of CD11b+ cells from acute slices and RT-PCR

Acute slices were obtained from C57BL/6J mice and allowed to rest for the time intervals as described above. The slices were cut to isolate the hippocampus, pooling together hippocampal regions from two mice. The hippocampal tissue was further mechanically dissociated using a glass wide-tipped pipette and the suspension was applied to a 30 μm cell strainer (Miltenyi Biotec). Cells were processed immediately for MACS MicroBeads separation. CD11b^+^ cells were magnetically labeled with CD11b MicroBeads. The cell suspension was loaded onto a MACS Column placed in the magnetic field of a MACS Separator and the negative fraction was collected. After removing the magnetic field, CD11b^+^ cells were eluted as a positive fraction. The purity of CD11b^+^ cell fraction was verified by immunofluorescence and flow cytometry (FACS) and was 99%” (Garofalo et al., 2017). After sorting the positive and negative fractions, total RNA was isolated with Trizol reagent, and processed for real-time PCR. The quality and yield of RNAs were verified using the NANODROP One system (Thermo Scientific). CD11b^+^ cells sorted from hippocampal slices were lysed in Trizol reagent for isolation of RNA. Reverse transcription reaction was performed in a thermocycler (MJ Mini Personal Thermal Cycler; Biorad) using IScript TM Reverse Transcription Supermix (Biorad) according to the manufacturer’s protocol, under the following conditions: incubation at 25°C for 5 min, reverse transcription at 42°C for 30 min, inactivation at 85°C for 5 min. Real-time PCR(RT-PCR) was carried out in a I-Cycler IQ Multicolor RT-PCR Detection System (Biorad) using SsoFast EvaGreen Supermix (Biorad) according to the manufacturer’s instructions. The PCR protocol consisted of 40 cycles of denaturation at 95°C for 30 s and annealing/extension at 60°C for 30 s. For quantification analysis, the comparative Threshold Cycle (Ct) method was used. The Ct values from each gene were analyzed in technical duplicates and normalized to the Ct value of *Gapdh* in the same RNA samples. Values differing by more than one Threshold Cycle (Ct) among each double were excluded. Relative quantification was performed using the 2-ΔΔCt method (Schmittgen and Livak, 2008) and expressed as fold change in arbitrary values. The cDNAs were amplified by PCR with specific primers: mouse *Tnf*α fw 5-GTGGAACTGGCAGAAGAG-3 and rev 5-CCATAGAACTGATGAGAGG-3, mouse *Il-1*β fw 5-GCAACTGTTCCTGAACTCAACT-3 and rev 5- ATCTTTTGGGGTCCGTCAACT-3, mouse *Cx3cr1* fw 5-TGACTGGCACTTCCTGCAGA-3 and rev 5-AGGGCGTAGAAGACGGACAG-3, mouse *P2y12r* fw 5-CACAGAGGGCTTTGGGAACTTA-3 and rev 5- TGGTCCTGCTTCTGCTGAATC-3.

### 2.7 Patch-clamp recordings from hippocampal pyramidal neurons

To study the effect of acute slicing on synaptic transmission, we performed a meta-analysis including previously published data (Zhan et al., 2014; Basilico et al., 2019a,2022b; Deivasigamani et al., 2023), classifying them according to the resting time before the cell was patched. As described above, the data were classified into two resting intervals: within 4 h after cutting or between 4 and 8 h after cutting. Acute slices from C57BL/6J were obtained as previously described. We recorded spontaneous excitatory postsynaptic currents (sEPSCs) and miniature excitatory postsynaptic currents (mEPSC) from hippocampal CA1 pyramidal neurons kept at −70 mV, using a patch clamp amplifier (Axopatch 200A, Molecular Devices, LLC). Data were filtered at 2 kHz, digitized (10 kHz), acquired with pClamp 10.0 software (Molecular Devices) and analyzed offline with Clampfit 10 (Molecular Devices, LLC). Patch pipettes (3–5 MΩ) were filled with an intracellular solution containing (in mM): Cs-methanesulfonate 135, HEPES 10, MgATP 2, NaGTP 0.3, CaCl_2_ 0.4, MgCl_2_ 2, QX-314 2, BAPTA 5 (pH adjusted to 7.3 with CsOH). Bicuculline methochloride (10 μM) was added to the extracellular solution to block GABAA receptors. mEPSCs were recorded after 10 min of bath perfusion with tetrodotoxin (TTX, 1 μM).

### 2.8 Dendritic spine density analysis

Images of the secondary and tertiary dendrites of the pyramidal neurons of CA1 *stratum radiatum* were acquired using a laser scanning confocal microscope (iX83 FV1200) with a 60x oil immersion objective. Fluorescence was detected by a photomultiplier in the EGFP channel using a 473nm laser. Confocal image stacks were acquired with 0.1 μm z-step, 1600 × 1600 pixels at 44 nm pixel size required for post-processing deconvolution. Image stacks were deconvoluted with the software Huygens Professional 18.04 software (parameters: SNR30, 50 iterations, 0.01 quality change). The analysis was performed using NeuronStudio and ImageJ softwares. We isolated segments of known length from secondary or tertiary dendrites and then reconstructed the segment with NeuronStudio software. Dendritic spines were automatically counted and undetected spines were added manually. To calculate the density of dendritic spines, the number of spines was divided by the length of the dendritic segment and expressed as the number per micrometer. Maximal intensity projections of the isolated segments were then opened with ImageJ and the diameter of the heads of all detected spines was measured.

### 2.9 Microglia-neuron contact density

The image stacks were acquired using a confocal laser scanning microscope (iX83 FV1200, Olympus) with an Olympus 60x oil immersion objective. Images were taken at the level of the CA1 *stratum radiatum*: in particular, images of neurons were taken at the level of the secondary and tertiary dendrites, the cell bodies of the neurons were not included. Fluorescence was detected in sequential mode using 473 nm and 559 nm lasers for EGFP and RFP channels respectively. Confocal image stacks were acquired with 0.2 μm z-step, 1600x1600 pixels at 53 nm pixel size required for post-processing deconvolution. Image stacks were deconvoluted with the software Huygens Professional 21.04 (SVI), using the following parameters: SNR 40, 50 iterations, 0.01 quality threshold.

Image stacks were visually inspected in ImageJ (National Institutes of Health, USA), to determine regions of co-localization of RFP-labeled microglia and GFP-labeled dendrites. First, we reconstructed the microglial domain, namely the volume occupied by a single microglial cell. Contacts within the microglial domain were determined by overlap between the red (microglia) and the green (neurons) channel, after independently determining the brightness for each channel. Identified overlaps were defined as contacts only if the red and the green channel had overlapping pixels in at least two Z-sections and if the overlap extended beyond four times the spatial resolution (pixel). Microglia-neuron contact density is reported as the number of contacts found in the microglial domain of a single microglial cell for field.

### 2.10 Statistical analysis

Origin 6 software was used for statistical analysis. Data were analyzed by unpaired *t*-test, when comparing only two groups, or one-way analysis of variance (ANOVA), when comparing more than two groups. Kolmogorov–Smirnov test was used to compare cumulative probability curves. Two-way ANOVA (time x voltage) was used to analyze the K^+^ microglial currents. Post Hoc comparisons were performed using the Holm–Sidak or Tukey test. All data are reported as mean ± SEM. The data of microglial morphology, dendritic spines density and head diameter and microglia-neuron contacts were normalized, expressed as percentages of the data obtained from the perfused tissue used as control. For electrophysiological recordings, n/N refers to cells/mice (whole-cell patch clamp).

## 3 Results

### 3.1 Microglia in acute slices show time-dependent changes in their morphofunctional properties, acquiring a reactive phenotype with time

**FIGURE 1:**
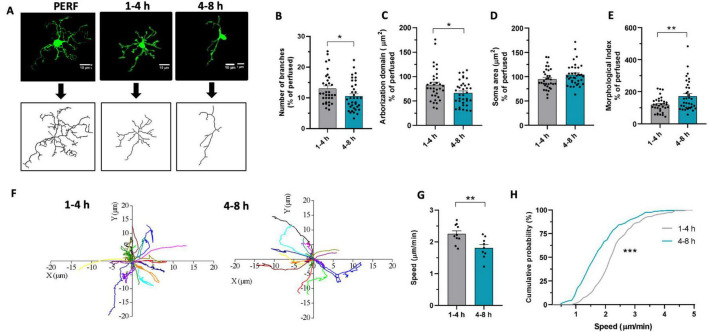
Microglia morphofunctional properties change over time in acute slices**. (A)** Representative z-stacks projections of GFP+ microglia (top) and reconstructed «skeleton» (bottom) from perfused slices (PERF) and from acute slices fixed after 1–4 h or 4–8 h of resting. **(B–E)** Quantitative analysis of microglia morphometry revealed reduced branches number after 4–8 h [**(B)**, *t*-test: *t* = 2.096, * *p* < 0.05], reduced arborization domain after 4–8 h [**(C)**, *t*-test: *t* = 2.372, * *p* < 0.05], no difference in soma area [**(D)**, *t*-test: *t* = 1.726, *p* = 0.089] and increased morphological index after 4–8 h [**(E)**, *t*-test: *t* = 2.896, ** *p* < 0.01]. 1–4 h *n* = 33 cells / 3 mice; 4–8 h *n* = 36 cells/3 mice. Data are shown as percentage of the mean value obtained in perfused mice. **(F)** Track analysis of microglial processes path after 1–4 h (left) or 4–8 h (right) measured through time-lapse fluorescence monitoring in acute slices. **(G)** Bar graph representing the speed of microglial processes after 1–4 h (gray, *n* = 150/10/3 processes, fields, mice numbers) and after 4-8h (green, *n* = 137/9/3). *T*-test: *t* = 3.09, ** *p* < 0.01. **(H)** Cumulative probability distribution of data represented in G. Kolmogorov – Smirnov test (*D* = 0.402, *** *p* < 0.001).

To assess whether microglia undergo morphofunctional changes during the hours following cutting, we first analyzed their morphology in acute slices of P40-50 *Cx3cr1*^*GFP*/+^ mice. For this purpose, 250-μm-thick acute slices were fixed in PFA 4% at two different time points: within 4 h after cutting (henceforth referred to as “*early*”) or between 4 and 8 h (henceforth referred to as “*late*”) after cutting. We reconstructed the tridimensional morphology of microglia from the CA1 hippocampal *stratum radiatum* at the different time points (Figure 1A). Morphological analysis revealed that *late* microglia display a lower number of processes (Figure 1B) and reduced arborization area (Figure 1C) compared to *early* ones. In addition, we observed an increase in the Morphological Index (soma area/arborization area) in the late phase compared to the early phase (Figure 1D), although without significant difference in the soma area (Figure 1E). These data suggest that in acute slices, within few hours from slicing, microglia acquire a less ramified morphology, occupying a smaller area in the tissue.

To assess the monitoring ability of microglia in the brain tissue, we compared the spontaneous motility of microglia processes at the chosen time points in *Cx3cr1*^*GFP*/+^ mice. Using time-lapse imaging, we monitored the motility of microglial processes for 20 min (Figure 1F). We observed a reduction in the speed of the movement of the processes in the late phase compared to the early phase (Figures 1G, H). This suggests that microglia exhibit time-dependent alteration of processes basal motility in acute slices, reinforcing the picture of reduced tissue monitoring capability in *late* microglia. A more complex pattern of time dependency emerged when considering microglia processes rearrangement induced by local ATP application (Pagani et al., 2015). As shown in Supplementary Figure 1, the time dependency of ATP-induced processes attraction did not easily fit in the early-late subdivision of this study. Our data indicate that the attraction of microglia processes caused by ATP application is minimal in the very early phase, reaching its peak in an ‘intermediate’ time window (2–4 h from cutting; Supplementary Figure 1), showing a tendency to slow down at later timing (4–6 h from cut).

**FIGURE 2:**
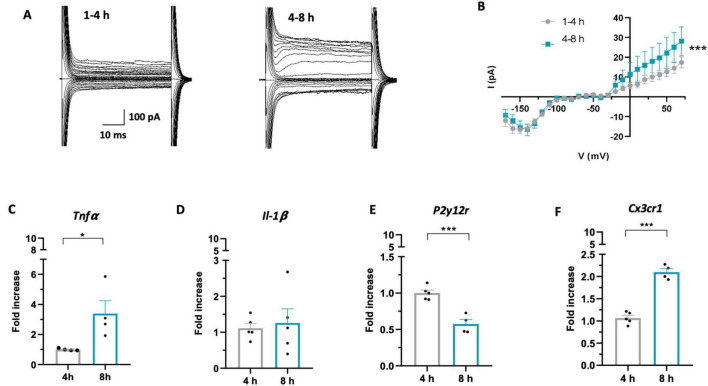
Microglia acquire a reactive phenotype over time in acute slices. **(A)** Representative traces of voltage-dependent currents recorded from microglia (from −170 to 70 mV; holding potential 70 mV), recorded after 1–4 h (left) or 4–8 h (right). **(B)** Average I–V curves from all recorded cells showing increased amplitude of outward K+ currents after 4–8h (green; *n* = 41 cells/12 mice) compared to 1–4h (gray; *n* = 61 cells / 14 mice) (Two-Way Anova: time *F* = 13.937; *** *p* < 0.001; potential *F* = 27.619, *p* < 0.001). **(C–F)** RT-PCR of the *Tnf*α **(C)**: 4h (gray; 8 mice) 8h (green; 8 mice). *T*-test *t* = 2.786, * *p* < 0.05), Il-1β **(D)** 4 h (gray; 10 mice) 8 h (green; 10 mice). *T*-test *t* = 0.352, *p* = 0.73), P2y12r **(E)**: 4h (gray; 10 mice), 8 h (green; 8 mice). *T*-test *t* = 5.831, *** *p* < 0.001), Cx3cr1 **(F)**: 4h (gray; 10 mice), 8 h (green; 8 mice) *T*-test *t* = 10.58, *** *p* < 0.001) gene in microglia isolated from acute slices after 4 h (gray) or 8 h (green). Note that in **(C–F)** each single data point represents a single extraction of microglia from 2 mouse brains pooled together.

Then, we investigated the changes in microglia electrophysiological properties depending on slices resting times, since voltage dependent K^+^ currents are known to be an indicator of phenotypic changes after brain disturbance (Boucsein et al., 2000). For this purpose, we performed a meta analysis of previously obtained recordings of outward and inward current response to voltage steps stimulation recorded from microglia in CA1 *stratum radiatum* of acute hippocampal slices from *Cx3cr1*^*GFP*/+^ mice (Pagani et al., 2015). Microglial cells recorded from acute slices in the two time-windows displayed both outward and inward voltage-dependent currents (Figure 2A). Interestingly, microglia recorded in the late phase displayed significantly higher amplitude of outward rectifying currents (IK) compared to cells patched in the early phase (Figure 2B), while no changes were observed in the inward component. The change in current amplitude was not accompanied by changes in microglia membrane passive properties (Supplementary Figure 2). An increase in amplitude of voltage-dependent K^+^ outward rectifying currents has been associated with reaction to tissue lesion or reactive microglia (Boucsein et al., 2000; Visentin et al., 2001; Avignone et al., 2008; Moussaud et al., 2009; Swiatkowski et al., 2016).

To confirm that microglia acquire a reactive phenotype during slice resting time, we analyzed the expression of specific proinflammatory cytokines and receptors relevant to microglia function in cells isolated from hippocampal slices in the two time windows. RT-PCR performed on isolated microglia revealed increased expression of TNF-α at 8 h after slicing compared to 4 h (Figure 2C), while no difference was observed in IL-1β expression (Figure 2D). Moreover, we found decreased expression of P2Y12 receptor (Figure 2E), usually defined as an “homeostatic” gene (Gómez Morillas et al., 2021) and increased expression of CX3CR1 receptor (Figure 2F), ligand of the neuronal chemokine CX3CL1.

Overall, these data indicate that in acute slices microglia undergo time-dependent morphological, functional and transcriptional changes, shifting towards a *reactive* phenotype with time.

### 3.2 Glutamatergic synaptic transmission changes in a time-dependent manner in acute slices

**FIGURE 3:**
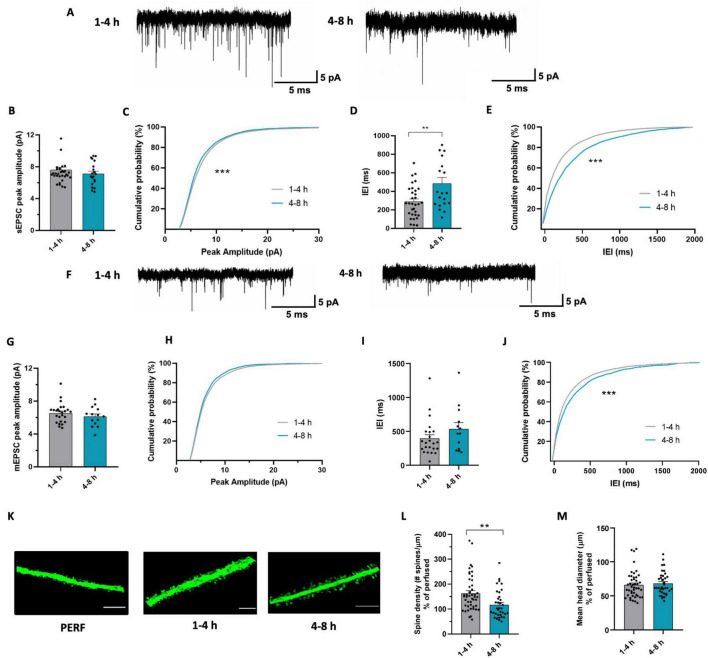
Glutamatergic synaptic transmission undergoes changes during time in acute slices. **(A)** Representative traces of sEPSC recorded at -70 mV from CA1 pyramidal neurons after 1–4 h (left) and after 4–8 h (right). **(B)** Bar graph of mean sEPSC amplitudes from cells patched after 1-4 h (*n* = 33 cells/18 mice) and after 4-8 h (*n* = 20 cells / 14 mice). *t*-test: *t* = 1.001; *p* = 0.3217. **(C)** Cumulative distributions of sEPSC amplitudes as in (b). Kolmogorov-Smirnov test: *D* = 0.169 *** *p* < 0.001. **(D)** Bar graph of mean sEPSC Inter-event interval from cells patched after 1–4 h (*n* = 32 cells/18mice) and after 4–8 h (*n* = 20 cells / 14 mice). *t*-test: *t* = 3.121, ** *p* < 0.01. **(E)** Cumulative distributions of sEPSC inter-event intervals as in (d). Kolmogorov-Smirnov test: *D* = 0.183, *** *p* < 0.001. **(F)** Representative traces of mEPSC recorded at –70 mV from CA1 pyramidal neurons after 1–4 h (left) and after 4–8 h (right). **(G)** Bar graph of mean mEPSC amplitudes from cells patched after 1–4 h (*n* = 25 cells / 13 mice) and after 4–8 h (*n* = 14 cells / 9 mice). *t*-test: *t* = 1.054, *p* = 0.2989. **(H)** Cumulative distributions of mEPSC amplitudes as in (g). Kolmogorov-Smirnov test: *D* = 0.035, *p* = 0.163. **(I)** Bar graph of mean mEPSC Interevent interval from cells patched after 1-4 h (*n* = 23 cells / 13 mice) and after 4-8 h (*n* = 13 cells/9 mice). *t*-test: *t* = 0.233, *p* = 0.816. **(J)** Cumulative distributions of mEPSC interevent intervals as in (I). Kolmogorov-Smirnov test: *D* = 0.13, *** *p* < 0.001. **(K)** Representative confocal images of dendritic segments belonging to pyramidal neurons in CA1 *stratum radiatum* of brain slices obtained from perfused mice (left) and acute slices fixed after 1–4 h (center) or 4–8 h of resting (right). Scale bar: 5 μm. **(L)** Bar graph of mean dendritic spines density of CA1 pyramidal neurons from acute slices fixed after 1–4 h (gray; *n* = 48/3, dendrites / mice) and after 4-8 h (green; *n* = 36/3). *t*-test: *t* = 3.174, ** *p* < 0.01. Data are shown as percentage of the mean value obtained in perfused mice. **(M)** Bar graph of mean spine head diameter in slices fixed after 1–4 h (gray; *n* = 48/3) and after 4–8 h (green; *n* = 36/3). *t*-test: *t* = 0.5189, *p* = 0.6053. Data are shown as percentage of the mean value obtained in perfused mice.

With the aim to understand whether synaptic transmission undergoes time-dependent changes in acute slices, we carried out a meta-analysis of three sets of recordings of glutamatergic spontaneous and miniature excitatory synaptic currents (sEPSCs and mEPSCs), obtained in similar conditions from pyramidal neurons of the CA1 area of the hippocampus (Zhan et al., 2014; Basilico et al., 2019a; Deivasigamani et al., 2023), by subdividing them in early and late, as described above. These experimental sets were chosen based on the following criteria: i) having similar distribution of EPSC peak amplitudes and ii) having a proper division into the two temporal categories. In these conditions, our data showed a reduction in sEPSC amplitude and frequency in the late time window (Figures 3A, B). In particular, EPSC amplitude reduction resulted from the comparison of cumulative sEPSC amplitudes distribution (Figure 3C), suggesting that in the late phase there is a higher probability of finding synaptic events with smaller amplitude than during the early phase. Similarly, the analysis of the interevent intervals (IEI) revealed a significant increase in IEI in cells patched in the late phase, indicative of event frequency reduction, as shown by both the average IEI (Figure 3D) and the cumulative distribution of sEPSC IEI (Figure 3E).

To further explore the impact of the resting time on synaptic transmission in acute slices, we compared the amplitude and frequency of mEPSCs recorded in the two time-windows (Figure 3F). We found no difference in the amplitude of mEPSCs (Figures 3G, H), but observed a reduction in mEPSC frequency in the late phase, as shown by the shift in the cumulative distribution of mEPSC IEI (Figures 3I, J). The time dependent decrease in both mEPSC and sEPSC frequency might be indicative of a decrease in the number of functional glutamatergic synapses during slice maintenance. To verify whether this functional alteration is supported by structural changes, we also analyzed the number of dendritic spines in acute slices fixed at the two different time points (Figure 3K). We observed reduced spine density in the late compared to the early phase (Figure 3L), which was not accompanied by differences in spine head diameter (Figure 3M). Note that data in Figure 3M are expressed as percentage of spine density in perfused mice. The observed decrease in dendritic spine density is consistent with the decrease in mEPSC frequency, suggesting a time-dependent decrease in the number of glutamatergic synapses (Kerchner and Nicoll, 2008) in slices.

Overall, these data suggest that synaptic transmission undergoes time-dependent changes after cutting, characterized by a general reduction of excitatory synaptic function based on the reduction of both the number and the strength of glutamatergic synapses.

### 3.3 Microglia mediate the time-dependent decrease in amplitude observed in glutamatergic synaptic transmission in acute slices

It is known that microglia are involved in glutamatergic synapse functioning and their removal or dysfunction has a profound effect on synaptic properties in mice (Basilico et al., 2019a,2022b). Thus, we hypothesized that the time-dependent changes in the morphofunctional properties of microglia could alter their ability to control synaptic function underlying time-dependent changes in synaptic transmission.

**FIGURE 4:**
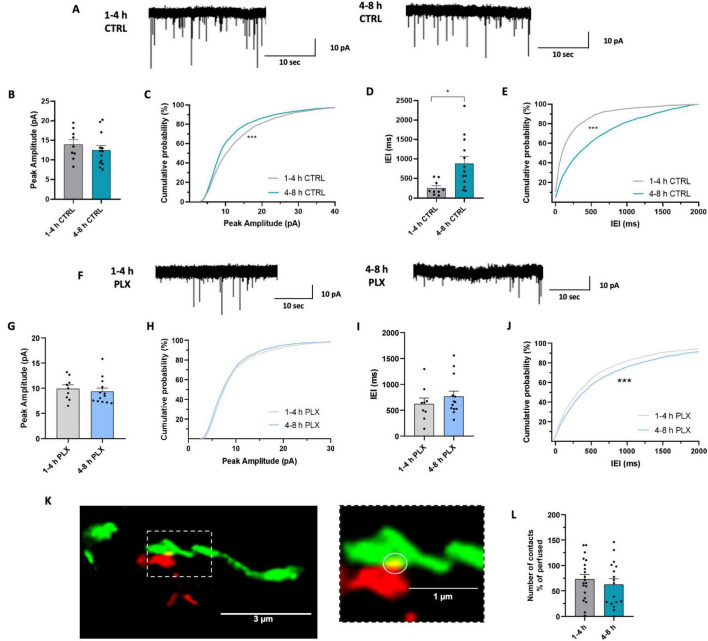
Microglia induce time-dependent changes in glutamatergic synaptic transmission in acute slices. **(A)** Representative traces of sEPSC recorded at –70 mV in CA1 pyramidal neurons from control mice after 1–4 h (left) and after 4–8 h (right) of resting. **(B)** Bar graph of mean sEPSC amplitudes from cells recorded after 1-4 h (*n* = 9 cells / 6 mice) and after 4–8 h (*n* = 13 cells / 4 mice) in control mice. *t*-test: *t* = 0.8227, *p* = 0.4204. **(C)** Cumulative distributions of sEPSC amplitudes as in (d). Kolmogorov-Smirnov test: *** *p* < 0.001; *D* = 0.12835. **(D)** Bar graph of mean sEPSC Interevent interval from cells recorded after 1–4 h (*n* = 9 cells / 6 mice) and after 4–8 h (*n* = 13 cells / 4 mice) in control mice. *t*-test * *t* = 2.778, *p < 0.05.* **(E)** Cumulative distributions of sEPSC interevent intervals as in **(F)**. Kolmogorov-Smirnov test: *D* = 0.376, *** *p* < 0.001. **(F)** Representative traces of sEPSC recorded at –70 mV in CA1 pyramidal neurons from microglia-depleted mice after 1–4 h (left) and after 4–8 h (right). **(G)** Bar graph of mean sEPSC amplitudes from cells recorded after 1–4 h (*n* = 9 cells / 6 mice) and after 4–8 h (*n* = 13 cells / 9 mice) in microglia-depleted mice. *t*-test *t* = 0.553, *p* = 0.59. **(H)** Cumulative distributions of sEPSC amplitudes as in (i). Kolmogorov-smirnov test *D* = 0.045, *p* = 0.07. **(I)** Bar graph of mean sEPSC Interevent interval from cells recorded after 1-4 h (*n* = 9 cells / 6 mice) and after 4–8 h (*n* = 13 cells / 9 mice) in microglia-depleted mice. *t*-test *t* = 0.923, *p* = 0.36. **(J)** Cumulative distributions of sEPSC interevent intervals as in (k). Kolmogorov-Smirnov test: *D* = 0.09048, *** *p* < 0.001. **(K)** Representative z-stack projection of a microglia-neuron contact, visualized as a yellow (circled) overlap between the red (microglia) and green (neurons) channels. Overlaps were considered as real contacts only if they were at least 4 contiguous pixels in size and if they were present in at least two consecutive z-stacks. The figure on the right is a zoom of the area within the dashed rectangle on the left. **(L)** Bar graph of mean number of microglia-neuron contacts in CA1 *stratum radiatum* from acute slices fixed after 1–4 h (gray; *n* = 20/3, microglial cells / mice) and after 4–8 h (green; 17/3). *t*-test: *t* = 0.7380; *p* = 0.4654. Data are shown as percentage of the mean value obtained in perfused mice.

To rule out the involvement of microglia, we investigated the time-dependent changes of hippocampal EPSCs properties in microglia-depleted mice (Basilico et al., 2022b). In this experimental set, mice were treated for 7 days with either a control diet or a diet containing PLX5622, a CSF1R inhibitor that induces a depletion of approximately 90% of microglia after one week, as previously quantified in Basilico et al., 2022b (Supplementary Figure 3; see also Elmore et al., 2014; Basilico et al., 2022a). We classified the data according to the two resting time categories described above (Figure 4A). According to results reported above, in slices from mice treated with the control diet, we observed a decrease of sEPSC amplitude in the late phase, as highlighted by the shift in the cumulative curve of sEPSC amplitudes (Figures 4B, C). In addition, we observed a decrease in frequency in cells recorded during the late phase compared with those recorded during the early phase (Figures 4D, E). Remarkably, in slices prepared from microglia-depleted mice, we found no time-dependent difference in the amplitude of sEPSCs (Figures 4F–H), indicating that time-dependent amplitude reduction requires microglia presence in the slice. Conversely, microglia depletion did not interfere with the reduction of synaptic frequency observed over time. In fact, in slices from PLX-treated mice, we observed a significant increase in the IEI of sEPSC during the late phase, as represented by the shift in the cumulative IEI probability curve (Figures 4I, J).

In order to evaluate whether acute slices cutting could be associated with time-dependent alterations of microglia-neuron interactions, we analyzed the density of contacts between microglia and neurons (Figure 4K), by fixing slices from Thy1::GFP; Cx3cr1::cre ERT2;R26CAG-tdtomato mice at different time points after cutting. As shown by the graph in Figure 4L, we found no significant difference in contact density between microglia and neurons in slices in the early and the late phase.

Taken together, these data indicate that microglia play a role in the time-dependent modulation of glutamatergic sEPSCs observed in acute slices. It has to be noted that not all functional synaptic changes happening in hippocampal slices are microglia-dependent. In fact, time dependent reduction in synaptic frequency is present also after microglia removal. The functional changes observed, however, are not accompanied by changes in microglia-neuron physical interactions, likely relying on different mechanisms.

## 4 Discussion

In this study, we investigated the effect of resting time after brain slices cutting on microglia and synaptic transmission. We observed that microglia undergo morphofunctional changes over time in acute slices, shifting to a reactive phenotype. Moreover, we observed alterations over time in glutamatergic synaptic transmission, with a decrease of both amplitude and frequency of spontaneous EPSCs. Remarkably, the amplitude decrease in glutamatergic sEPSCs observed over time was microglia-dependent.

### 4.1 Microglia morphology and motility changes over time in acute slices

We report here that both morphology and motility of microglia undergo time-dependent alterations during hours after slice cutting. In particular, we observed a decrease in the number of processes and a reduction in the arborization domain of microglia in slices during the late phase; concomitantly, we also observed a decrease in the speed of microglia processes’ movement. Altogether, our results show that during the resting time after cutting, microglia undergo a phenotypic shift; microglia in the late phase occupy a smaller volume in the brain parenchyma and their processes move slower, suggesting a time-dependent reduction in the patrolling activity.

A few studies have similarly reported that, over time, microglia acquire a less branched, amoeboid phenotype both in cultured (Stence et al., 2001) and acute slices (Matyash et al., 2017; see also Berki et al., 2024). In particular, Berki et al. (2024) recently demonstrated that microglia in slices undergo rapid morphological changes. In spite of the different time windows considered, these observations strongly support our findings of reduction of microglia branching during slice resting time. Morphological changes have been long associated with microglia reactivity, although the observed change in morphology itself does not necessarily describe a transition to a reactive phenotype (Paolicelli et al., 2022). Still, this finding, along with those discussed below, strengthens the idea that microglia are shifting towards a reactive phenotype. Indeed, it is now known that reactive microglia can assume different functional profiles (‘amoeboid’ microglia, ‘phagocytic’ microglia, ‘primed’ microglia) and in many of them there is a retraction of the processes (Paolicelli et al., 2022). In addition, a correlation has been reported between reduced microglial branching and increased release of the proinflammatory cytokine IL-1β (Madry et al., 2018). Therefore, these morphological alterations should be considered as cues that encourage further exploration of the correlation between morphological and functional changes in microglia using additional techniques.

Processes motility of microglia cells has attracted considerable interest in the last years, being strongly associated with microglia functions and abilities (Nimmerjahn et al., 2005; Wake et al., 2009; Andoh and Koyama, 2021b). It is known that two different types of motility exist: a motility for tissue surveillance and one for cell damage resolution (Davalos et al., 2005; Nimmerjahn et al., 2005). The first type of motility is associated with the activity of the K^+^ two-pore domain channel THIK-1, which is also involved in maintaining the resting potential of microglia (Madry et al., 2018). The second depends on the P2Y12 receptor (P2Y12R) that binds ATP and ADP (Davalos et al., 2005). Noticeably, we observed that both basal and ATP-induced motility of microglia processes are altered during slice resting time. We speculate that these changes may both depend on microglial changes and slice conditions, including, for example, changes in the level of purines as a result of slicing and time of rest (Duan et al., 2009; Matyash et al., 2017; Andoh and Koyama, 2021a). In particular, the complex pattern of variation of ATP-induced processes rearrangement could be ascribed to several factors, including the high ATP levels after slicing (Berki et al., 2024) and to microglia changes in branching and P2Y12R expression, all influencing microglia sensitivity to exogenous ATP (Pagani et al., 2015, 2020). Concerning the analysis of processes basal speed, it has to be pointed out that it does not take into account the direction in which the processes moved with respect to the cell body, i.e. whether they extended or retracted (Pagani et al., 2015, 2020; Basilico et al., 2019a; Andoh and Koyama, 2021a; Cordella et al., 2021). However, the dynamic of extension and retraction of processes is a key property that modulates microglial functions: production and release of inflammatory mediators, phagocytosis and contacts with synapses. Noticeably, it has been reported in THIK1 KO mice that reduced basal motility is accompanied by a reduction in microglial cell arborization (Madry et al., 2018), highlighting a correlative relationship between motility and microglial morphology. We speculate that these morphofunctional changes could alter the physiological activity of these cells over time in acute slices.

### 4.2 Microglia acquire a reactive phenotype over time in acute slices

The time dependent transition of slice microglia to a reactive phenotype is supported by functional and PCR data. Indeed, we observed an increase in K^+^ voltage-dependent outward currents in microglial cells recorded during the late compared to the early phase. On the contrary, we observed no difference in the amplitude of inward K^+^ currents between the early and late phases. Changes in the amplitude of microglia voltage-dependent K^+^ currents have been observed to occur in various experimental contexts, such as in pathological or inflammation models. In particular, in cultured microglia, characterized by large inward rectifying K^+^ currents (Kettenmann et al., 1990), inflammatory stimuli, such as LPS, ATP or TNFα, induce the activation of K^+^ outward currents (Pyo et al., 1997; Li et al., 2008; Nguyen et al., 2017; McLARNON et al., n.d.). Compared to microglia in culture, “resting” microglia in acute slices from adult mice show little or no voltage-dependent conductances. However, these currents are present in acute slices from developing mice, particularly during the second and third postnatal weeks (Pagani et al., 2015; Schilling and Eder, 2015), possibly in association with the intense synaptic turnover typical of this period (Izquierdo et al., 2021). Noticeably, the presence of outward rectifying K^+^ currents in acute slices from adult mice is associated with pathological or inflammatory patterns. In particular, outward rectifying K^+^ currents appear hours following nerve lesion (Boucsein et al., 2000; Gu et al., 2022), suggesting that these currents are a hallmark of a transition towards a reactive phenotype. Recent work has indeed revealed that outward K^+^ currents are essential for pro-inflammatory microglial activation in vivo and the subsequent release of pro-inflammatory cytokines such as TNFα and IL-1β (Di Lucente et al., 2018).

Gene expression analysis by RT-PCR of microglia isolated from acute slices revealed time-dependent changes in the expression levels of key microglial genes, supporting the notion of the transition to reactive phenotype. Indeed, after 8 h we observed an increase in the expression of *Tnf*α and *Cx3cr1*, a decrease in *P2y12* receptor, while no changes in *Il-1*β expression. In particular, the increase in the pro-inflammatory cytokine TNFα is a marked indicator of microglia reactivity; this cytokine modulates and assists the inflammatory response by inducing the secretion of other cytokines by microglia (Kuno et al., 2005) and by promoting the pro-inflammatory activity of astrocytes (Raffaele et al., 2020). Furthermore, it is important to note that TNFα is also a recognized modulator of synaptic function and changes in its expression could lead to alterations in neuronal synaptic transmission (Pickering et al., 2005; Stellwagen and Malenka, 2006; He et al., 2012).

Normally, neurons express the chemokine CX3CL1, which interacts with its receptor on microglia, namely CX3CR1. The constant CX3CL1/CX3CR1 interaction under physiological conditions has been shown to contribute to maintaining microglia in homeostatic conditions (Limatola and Ransohoff, 2014). CX3CR1 expression level has been shown to increase in the presence of proinflammatory stimuli such as TNFα and IL-1β (Maciejewski-Lenoir et al., 1999) and in various disease models in which microgliosis occurs (Zhang et al., 2018; González-Prieto et al., 2021).

P2Y12 receptor is necessary for microglial branches movement towards sites of damage in which ATP and its hydrolytic products are released (Madry et al., 2018; Illes et al., 2020). It has been described that when ATP is released into the extracellular space by CNS injured cells, microglia undergo a biphasic variation in P2Y12 receptor expression: a first phase of increased expression in which microglia move their processes towards the site of injury and a second phase of reduced expression in which microglia retract their processes and begin the transition to a reactive phenotype (Orr et al., 2009; Koizumi et al., 2013; Illes et al., 2020). We speculate that the observed decrease in microglial P2Y12R expression during the late phase might be indicative of microglia shift to this second phase. This interpretation is consistent with the rest of our results, pointing to microglia shifting towards an activated phenotype during resting time. Of note, a time-dependent decrease in P2Y12R immunoreactivity has been recently reported in acute slices by Berki et al. (2024). These authors also pointed out that P2Y12R signaling is relevant in microglia remodeling in this model. However, our results are not easily comparable due to the different time periods considered.

Although increased expression of IL-lβ is a characteristic of reactive microglia (Basu et al., 2004; Kettenmann et al., 2011), we found no change in the expression of this cytokine over time after acute slices cutting. Importantly, microglia do not switch from precisely defined ‘resting’ to ‘activated’ state. It is now accepted that microglia in the living brain do not polarize into one of these categories, but can assume a variety of phenotypes depending on the functional contexts it encountered, often co-expressing pro- and anti-inflammatory markers (Paolicelli et al., 2022). Based on the published literature, the lack of an increase in IL-1β, despite the concomitant rise in K+ voltage-dependent outward currents in our dataset, might seem like a discrepancy. However, it is important to consider that the LPS stimulus, which induces IL-1β release in slices (Madry et al., 2018; Di Lucente et al., 2018), likely results in a stronger effect than the events we observe in acute slices during the resting state. This evidence suggests that a careful analysis of the expression of different markers is necessary to more accurately describe the phenotype assumed by microglia in the acute slices. Since, our analysis by RT-PCR was limited to a small subset of genes important for microglial function, further investigations would be necessary in order to more accurately describe the phenotype assumed by microglia in the late phase. It should be also noted that the RT-PCR data here reported are based on specific time points rather than time windows, as the specimens were collected at 4h and 8h, referring only to the end of those periods, not the entire time span. Therefore, caution is required when comparing these data with those based on entire time windows. Additionally, the data represent relative variations over time but do not indicate the baseline condition, and may not capture changes occurring on a faster time scale, as those reported by Berki et al. (2024).

### 4.3 Glutamatergic synaptic transmission undergoes time-dependent changes in acute slices

In order to assess whether glutamatergic synaptic transmission in the hippocampus of acute slices undergoes time-dependent modifications, we compared over time the properties of sEPSCs and mEPSCs from CA1 pyramidal neurons in comparable experimental sets, observing a decrease in the frequency of both sEPSCs and mEPSCs and in sEPSCs amplitude in the late phase.

The reduction in mEPSC and sEPSC frequency is likely indicative of a decrease in the number of functional synapses (Turrigiano and Nelson, 2004; Segal, 2010). In this respect, it should be considered that mEPSCs represent a significant fraction of sEPSCs (Hsia et al., 1998), and they are reasonably responsible for the reduction in frequency of the latter. Time dependent reduction in functional excitatory synapses is in accordance with the observed reduction of dendritic spines density observed in the late phase. Several studies have reported that acute slices have higher dendritic spines density than perfused brain slices (Kirov et al., 1999; Trivino-Paredes et al., 2019), suggesting that synaptogenesis is occurring in acute slices (Kirov et al., 1999). More recently, a work described in acute slices a population of thalamocortical synapses representing newly-formed synapses (Crocker-Buque et al., 2015). Interestingly, the amount of these newly formed synapses decreases 2 h after slicing and it was shown by the authors that this is due to microglia-mediated synaptic elimination (Luz et al., 2017). This evidence suggests that in the first hours, synaptogenesis occurs in acute slices and subsequently, synapses that have not been stabilized are eliminated (Luz et al., 2017). This is in agreement with our findings and suggests the possibility of time-dependent structural changes in synapses, pointing out that the time spent in vitro by the slice is a significant variable in experiments evaluating synaptic transmission in acute brain slices.

Unlike control animals, microglia-depleted mice lack the observed difference in the amplitude of sEPSCs between the early and late phase, suggesting that the decrease in sEPSC amplitude in the late phase is microglia-dependent. We have previously reported that microglia modulate synaptic transmission in the hippocampus of adult mice and that in the absence of microglia-neuron crosstalk, the glutamatergic activity of hippocampal CA1 pyramidal neurons is depressed, with weaker and defective synapses and reduced sEPSC amplitude (Basilico et al., 2022b). The meta-analysis of the data obtained in the same study, revealed that in the absence of microglia, there is no difference in sEPSC amplitude between the early and late phases. This finding suggests that under normal conditions, microglia might be involved in time-dependent modulation of synapses in the acute slice.

It is currently known that microglia modulate synaptic functioning not only constitutively (Badimon et al., 2020; Yegla et al., 2021; Basilico et al., 2022b), but also as a consequence of the alteration of the brain tissue homeostasis by the presence of acute stimuli (Tremblay et al., 2010; Pascual et al., 2012; Riazi et al., 2015). As extensively described in literature, the mechanisms by which microglia modulate synapses are mainly two: physical contact of synapses (Miyamoto et al., 2016; Akiyoshi et al., 2018; Weinhard et al., 2018; Cserép et al., 2021) and the release of diffusible substances (Lauro et al., 2006; Pascual et al., 2012; Parkhurst et al., 2013; Lin et al., 2019; Pinto et al., 2023). We observed that the density of contacts between microglia and neurons does not show time-dependent changes, regardless of reduction of microglia branching. On one hand, this suggests that the interaction sites are somehow privileged or protected; on the other hand, it highlights that the modulation of synapses by microglia during resting time may rely on other mechanisms, such as the release of neuromodulatory substances. The increase of TNFα expression in microglia obtained through rtPCR suggests that one possible modulator may be TNFα, which is a known synaptic modulator involved in several models of microglia-dependent synaptic modulation (Pickering et al., 2005; Yamamoto et al., 2019; Raffaele et al., 2020; Wegrzyn et al., 2021).

Interestingly, the time-dependent reduction in the frequency of sEPSCs was also observed in mice deprived of microglia, suggesting that this change in frequency is not due to microglia but to other factors yet to be elucidated. Over time, structural changes in the circuits could occur, as also reported by Berki et al. (2024), leading to a reduction in frequency; alternatively the reduction in frequency could be due to metabolic factors (Segal, 2010; Ivanov and Zilberter, 2011; Nomura et al., 2019). However, it cannot be excluded the existence of multiple mechanisms affecting synaptic properties in parallel. We speculate that frequency reduction could be partially microglia-dependent and occluded by changes occurring in the early phase in the absence of microglia.

Regarding the electrophysiology data discussed above, it is particularly important to describe the procedure by which our meta-analysis was conducted. The data included, although from different experimental sets (Zhan et al., 2014; Basilico et al., 2019a; Deivasigamani et al., 2023), are consistent in terms of values of amplitude and frequency observed and methodology by which they were obtained. Furthermore, the decrease in amplitude and frequency of sEPSCs was obtained in a first dataset (Figures 3B–E) but also in an independent set of data, representing the control group of microglia-depleted mice (Basilico et al., 2022b; Figures 4B–E), corroborating the reliability of these data.

### 4.4 Methodological limitations and metabolic aspects

When discussing our results, it is necessary to consider two fundamental aspects in the analysis and evaluation of data obtained from acute slices: i) the method by which the slices are prepared and ii) the metabolic aspects involved. The most common method of cutting acute slices involves the use of ice-cold ACSF solutions in which sodium chloride (NaCl) is replaced with an inert substance in order to protect cells from swelling following cutting (Moyer and Brown, 1998; Mainen et al., 1999; Ye et al., 2006; Tanaka et al., 2008). In fact, during slicing, the passive influx of sodium leads to the subsequent entry of water and swelling of the cells. This event is the main cause of the poor survival of neurons, particularly those located in the superficial layers of slices. Another modification of the cutting procedure that is often used is to perform a short recovery period at 35-37°C to recover neuronal activity before electrophysiological recordings (Bischofberger et al., 2006; Ting et al., 2014). However, some authors suggest performing the entire procedure at physiological temperature (Ankri et al., 2014; Eguchi et al., 2020). Several authors have indeed observed that the use of cold solutions can alter microtubules and dendritic spines (Fiala et al., 2002; Kirov et al., 2004; Eguchi et al., 2020) and reduce the expression of AMPA receptor subunits (Taubenfeld et al., 2002). The methodology to obtain acute slices remains a critical issue and the data just discussed highlight the attention required in choosing and carrying out the dissection and cutting procedure. With regard to the work we conducted, it is important to note that Berki and colleagues also observed a time-dependent progression of microglia toward a reactive state and repeated their experiments by changing the solution and cutoff temperature and similarly observed microglial reactivity (Berki et al., 2024). Equally important is the environment in which the slices are allowed to recover. Buskila et al. (2014) introduced the Braincubator system as an alternative to the common submerged recovery chambers, such as the one used in our experimental setup. They demonstrated that the Braincubator is effective in maintaining the viability of acute brain slices for up to 36 h. In the context of our experiments, all analyses were conducted within 8 h of slice preparation, a timeframe during which bacterial growth remains controlled and cell viability is only slightly affected. Among the potential limitations, it should be also considered mice anesthesia, that might represent an additional confounding factor in interpreting time-dependent changes in slices. In particular, it is known that the acute application of volatile anesthetics in slices can alter synaptic and microglial properties (Madry et al., 2018; Kitamura et al., 2003).

Regarding the metabolic aspect, little is known about how the availability of energy substrates affects neuronal excitability. A crucial role is played by the oxygen rate provided to the slice: brain slices are supplied with gas and nutrients exclusively through the ACSF, with typical flows of 2-3 ml/min. Neuronal network oscillation has been shown to be closely related to the rate of oxygen supplied to acute slices: only a flow rate of 15 ml/min of oxygen preserves network activity (Ivanov and Zilberter, 2011). This implies that special care must be taken when choosing perfusion systems and incubation chambers. One question that might be important to raise is how closely the concentrations of energy substrates should mimic those found in the brain in vivo. It is clear that, in most cases, slicing involves radical changes in energy substrates that alter the process of energy homeostasis that occurs in vivo. This implies that, in acute slices, energy substrates must be supplied in higher concentrations than those present in the brain in vivo (Zilberter et al., 2010). In addition, some metabolic substrates important for brain function, such as lactate and ketone bodies, are not present in ACSF and therefore are not supplied to acute brain slices. The only energy substrate normally supplied through ACSF is glucose, which may not be sufficient to fully mimic the brain activity that occurs in vivo. In fact, it was observed that replacing standard ACSF with ACSF also containing ketone bodies and lactate induced a radical hyperpolarization of both the resting membrane potential and the Cl^–^ reversal potential (Holmgren et al., 2010), suggesting that energy defects could lead to neuronal hyperactivity. What has been described so far suggests that alterations in neuronal function caused by cutting procedures are unavoidable. However, in many cases these alterations could be avoided by aiming to reproduce the biochemical microenvironment surrounding neurons in an intact brain.

Overall, the evidence discussed above indicates that in acute slices, microglia undergo time-dependent modifications and that these changes are reflected in synaptic transmission through mechanisms yet to be elucidated. Importantly, synapses change their function and structure during experiments and further characterization is certainly needed. We conclude that microglia play a significant role in these changes, which can be considered forms of plasticity in acute slices, potentially modeling natural phenomena. Notably, this raises the critical issue of defining stable conditions for synaptic functioning in order to establish experimental protocols within time windows where there are no fluctuations in the properties being studied.

## Data Availability

The raw data supporting the conclusions of this article will be made available by the authors, without undue reservation.
