# Supplementary material for: Time-dependent phenotypical changes of microglia drive alterations in hippocampal synaptic transmission in acute slices
Source: Front Cell Neurosci. 2024 Nov 15;18:1456974. doi: 10.3389/fncel.2024.1456974 (PMC11604457; doi:10.3389/fncel.2024.1456974)
Supplement: Supplementary file 1 [file Data_Sheet_1.docx]

***Supplementary Material***

## Supplementary Figures


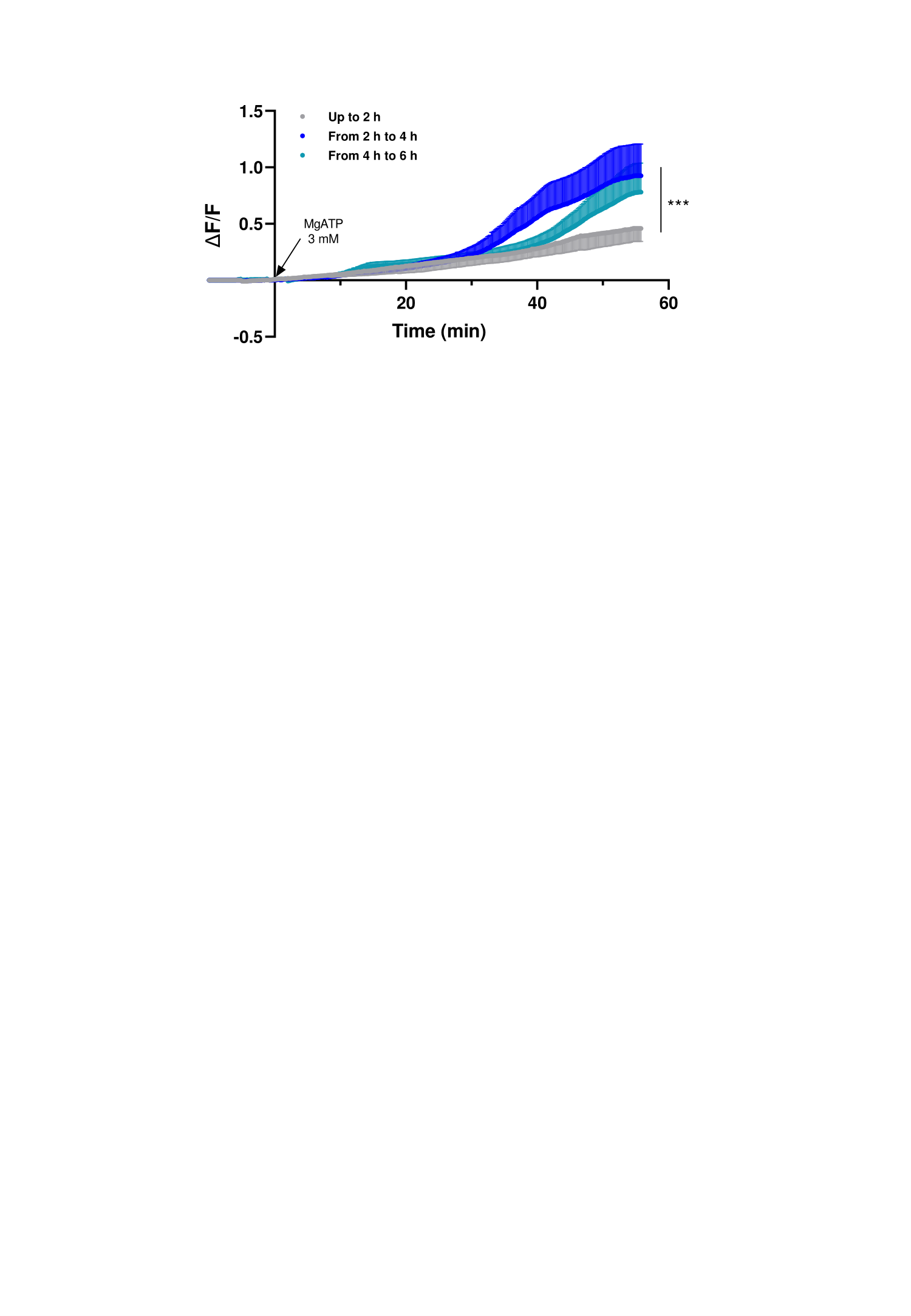


**Supplementary Figure 1: ATP-induced microglia processes rearrangement undergoes time-dependent changes in acute slices.** Time course of fluorescence ratio signal (DF/F0) measured in a circle (10 μm radius) centered on the tip of the ATP-containing pipette, from acute slices from Cx3cr1^+/gfp^ mice up to 2 hours (n = 7 / 6, slices / mice), from 2 to 4 hours (n =7 / 6) and from 4 to 6 hours (n=10 / 8) after slicing (2 way-Anova: p<0,001, F1 (time after cutting) = 0.00414E-7).


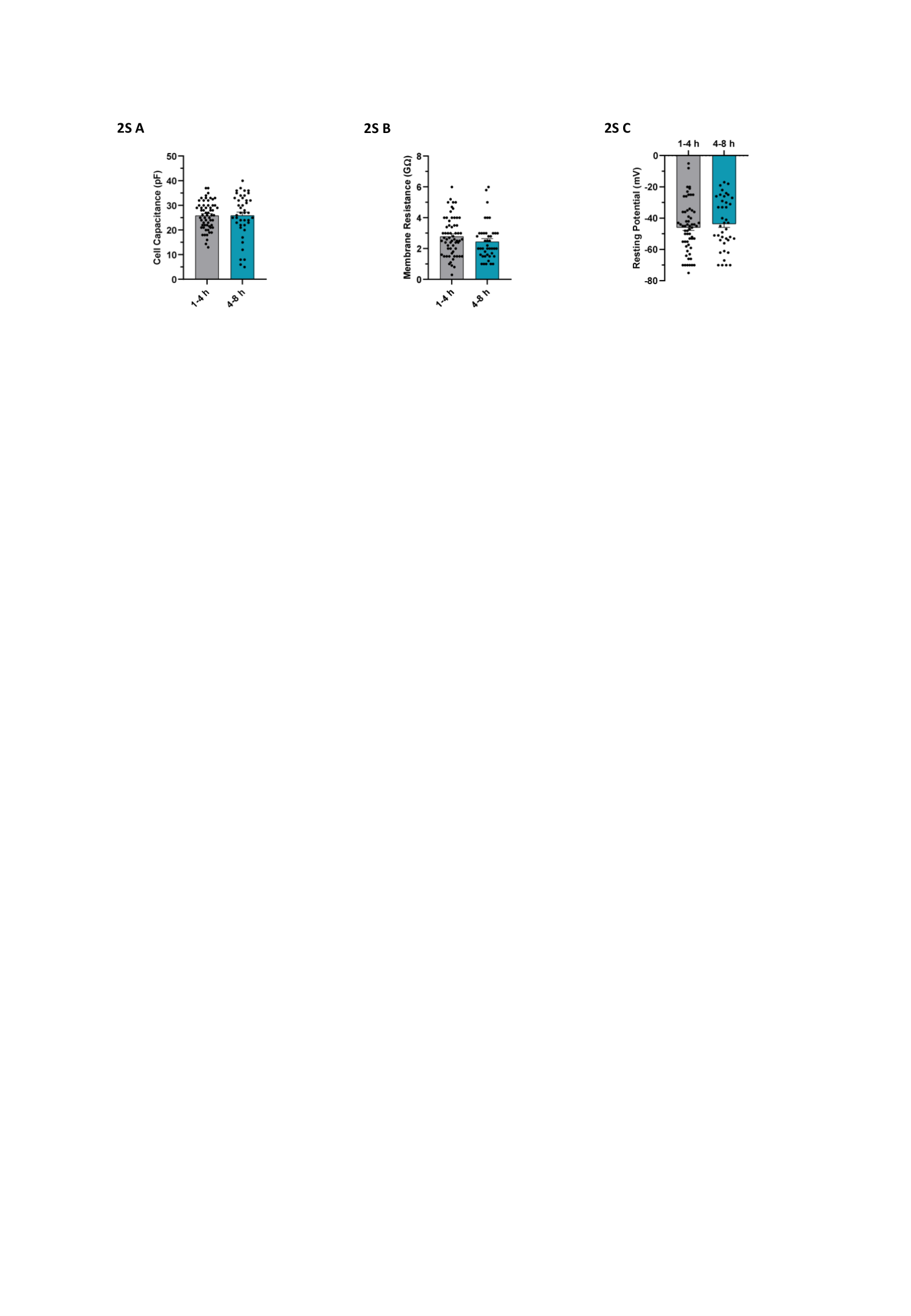


**Supplementary Figure 2: Microglia passive properties remain stable over time after acute slices cutting. 2S A:** Bar graph of mean cell capacitance of microglial cells recorded after 1-4h (grey; n = 60 cells / 14 mice) and 4-8h (green; n = 42 cells / 12 mice). T-test: t = 0.015, p = 0.9878. **2S B:** Bar graph of mean membrane resistance of microglial cells recorded after 1-4h (grey; n = 62 cells / 14 mice) and 4-8h (green; n = 42 cells / 12 mice). T-test: t = 1.399, p = 0.165. **2S C:** Bar graph of mean resting potential of microglial cells recorded after 1-4h (grey; n = 60 cells / 14 mice) and 4-8h (green; n = 40 cells / 12 mice). T-test: t = 0.7230, p = 0.4714.


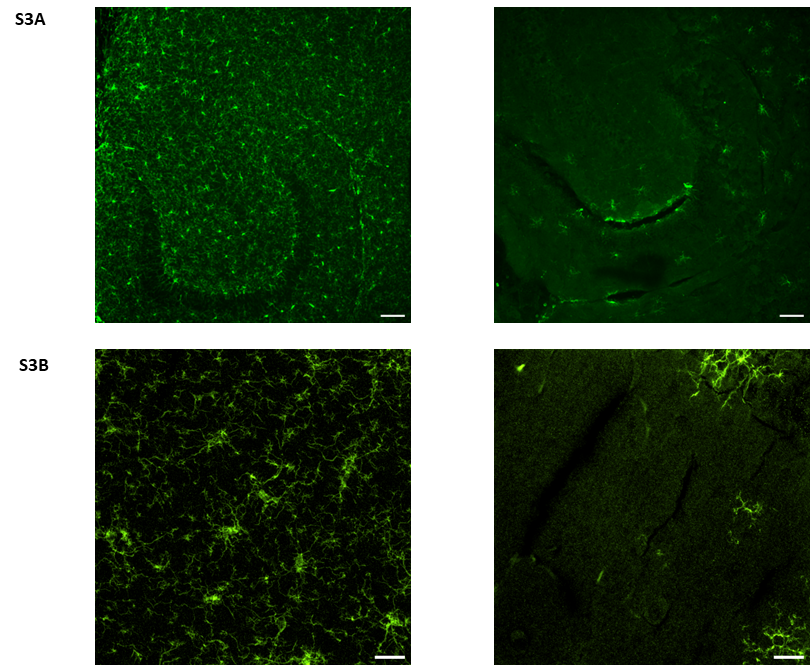


**Supplementary Figure 3. PLX5622 treatment induces microglial depletion.**

**3SA:** Representative confocal images of Iba+ microglial cells in the hippocampus, acquired with a 10x objective (scale bar: 100 µm), in control (CTRL, left) and PLX-treated mice (right). **3SB:** Confocal images of the hippocampal *stratum radiatum* of CTRL (left) and PLX-treated mice (right), acquired with a 40x objective (scale bar: 20 µm).
